# Nomad scientists and the ones left behind

**DOI:** 10.7554/eLife.30183

**Published:** 2017-07-11

**Authors:** Maya Bar, Barak Rotblat, Oded Rechavi

**Affiliations:** 1 Department of Plant Pathology and Weed Research, Agricultural Research Organization, Volcani Center, Rishon LeZion, Israel; 2 Department of Life Sciences and the National Institute for Biotechnology in the Negev, Ben-Gurion University of the Negev, Beer-Sheva, Israel; 3 Department of Neurobiology, Wise Faculty of Life Sciences and the Sagol School of Neuroscience, Tel Aviv University, Tel Aviv, Israel

**Keywords:** point of view, careers in science, mobility, postdocs, women in science, diversity

## Abstract

To improve the diversity of the scientific workforce, we should not penalize researchers who are unable to move abroad for long periods.

Studies confirm the intuitive assumption that being 'mobile' and following one’s dreams across borders is correlated with academic success (Veugelers, 2017). However, while the scientific community is making efforts to promote healthier work-life balances and to improve gender equality, the competitive path that leads to permanent research positions requires young scientists to uproot their lives at the exact time when most are engaged in starting their families. The concept of mobility has become so sacred in some fields that it is almost impossible to climb the steps of the academic ladder without relocating to a different country. Those who cannot leave, often women, are frequently dropped from the system as a result.

In our home country, Israel, and in many other countries, completing long, multi-year postdoctoral fellowships abroad is a prerequisite to landing a principal investigator position. Equally fruitful training (in terms of research output) in an otherwise excellent local lab rarely suffices. In general, continuing one’s studies in the same institute, city or country is considered CV sabotage.

In 2013, in an essay titled “Crossing oceans”, Eve Marder equated scientists who take risks and go abroad to early explorers, whose voyages “shaped their views of the world, and the tales they told expanded the universe for all those they encountered” (Marder, 2013). Indeed, the advantages of crossing oceans are undeniably many. A postdoc abroad is an opportunity to prove your independence, to discover other cultures, and to follow the lead of the very best, wherever they may be. Moreover, networking establishes connections that are often invaluable later on.

It is widely acknowledged that it is difficult for many researchers to displace their spouses and children to another country or continent. However, institutions and scientists in positions of power can sometimes be heard voicing the view that “those who really want it, will manage somehow”. The willingness of a researcher to make this sacrifice is often seen as evidence of their commitment, willpower and worthiness.

The issue is not restricted to postdoctoral training overseas. Taking mobility for granted is now hardwired into the structure of the academic system, and young principal investigators are often hired on temporary contracts lasting just a few years. From the point of view of an institution, constantly reshuffling the staff ensures that the research stays 'fresh', and that the researchers keep performing at the highest competitive level.

Forcing young researchers to move abroad based on these considerations does not take into account the personal lives of the scientists that have to “move on”. Aside from the financial shock (families living on the salary of one postdoc will live in relative poverty), the spouse often has to put on hold their dreams and aspirations. Such a situation would be a difficult test for any relationship. Raising children abroad, without the social and family safety nets that exist at home, can also be stressful, particularly if the children or spouse do not initially speak the local language. This may discourage parents from moving, especially as many are justifiably unwilling to make compromises when it comes to their children’s upbringing.

All these hardships affect women scientists the most. Women are underrepresented in most scientific fields, especially in principal investigator positions in academic institutions. The gender bias in science is a complex problem that is currently under investigation. While it seems that some of its root causes begin as early as grade school (Bian et al., 2017), we argue that mobility is one of the major bottlenecks that drives the gender disparity. Reports indicate that while roughly equal numbers of men and women complete PhDs in the life sciences, far more men than women move to a foreign country for postdoctoral training (NPA ADVANCE, 2001). In our opinion, a comprehensive effort to remedy the gender bias in the top tier of science needs to address the mobility issue head on.

The gender problem will not be solved until mobility is no longer an issue

Many academic institutions have established ad hoc committees to address gender bias. Some of the ideas that have been implemented include policies allowing for career breaks, and fellowships supporting women who have taken such breaks. In some cases, fellowships are provided to support the spouses of women postdocs during their stay abroad. Some institutions prioritize housing for visiting families and offer day care services. While these policies can be helpful in specific cases, we argue that such measures will not solve the fundamental problem – the gender and immigration-related issues that prevent many scientists from leaving their home country.

Therefore, we have one simple and straightforward message: it should not be an absolute requirement to do a postdoc abroad. Training overseas should be considered a plus, but not a prerequisite. Scientists should be judged solely based on their achievements. We argue that the gender problem will not be solved until mobility is no longer an issue. Furthermore, taking the difficulties that moving countries entails into account may help us to understand the stress typically experienced by researchers early in their scientific careers (Pain, 2017; The Graduate Assembly, University of California, Berkeley, 2014), and help us to convince the most talented of the young generation to join our profession.

The importance of mobility was ingrained into our minds many years ago, before globalization, when it was much harder to communicate and collaborate with international colleagues. Now we have email and video conferencing. Many of the advantages of long-term stays abroad could be gained by short visits and by attending international conferences. Today, there are great researchers all over the world, and one can find a top-notch lab in which to train pretty much everywhere.

In summary, we urge policy makers, job search committees, and grant agencies to re-assess the significance given to long-term stays abroad, given the heavy price paid by individual scientists and the scientific community as a whole. There are important measures being taken to support the family life of scientists, and to address the gender bias in academia. Eliminating one of the major underlying issues, the requirement to travel, could reduce the need to take affirmative action later on, and could allow a more diverse group of scientists to pursue academic careers.
